# *Chromobacterium sp.* septicemia in Sweden. A clinical case report

**DOI:** 10.1186/s12941-024-00692-5

**Published:** 2024-04-18

**Authors:** Oscar Backrud, Erik Engberg, Kristina Nyberg, Peter Wieslander, Edward R. B. Moore

**Affiliations:** 1 Department of Infectious Diseases, Sundsvall Hospital, Sundsvall, Sweden; 2 Department of Medicine Solna, Karolinska Institutet, Solna, Sweden; 3 Department of Laboratory Medicine, Sundsvall Hospital, Sundsvall, Sweden; 4 Department of Infectious Diseases, Institute for Biomedicine, Sahlgrenska Academy, University of Gothenburg, Gothenburg, Sweden; 5 Culture Collection University of Gothenburg (CCUG), Sahlgrenska Academy, University of Gothenburg and Sahlgrenska University Hospital, Gothenburg, Sweden; 6 Department of Clinical Microbiology, Sahlgrenska University Hospital, Region Västra Götaland, Gothenburg, Sweden

**Keywords:** Chromobacterium, Novel species, Chromobacterium violaceum, Infection, Septicemia, Case report, Sweden

## Abstract

**Background:**

*Chromobacterium* is a genus of fourteen species with validly published names, most often found in soil and waters in tropical and subtropical regions around the world. The most well-known species of the genus, *C. violaceum*, occasionally causes clinically relevant infections; cases of soft tissue infections with septicemia and fatal outcomes have been described.

**Case presentation:**

Here, we present a clinical case report of a 79-year-old man from Sweden with a soft-tissue infection and septicemia. The pathogen was identified as a strain of *Chromobacterium* species, but not *C. violaceum*. The patient was treated with clindamycin and ciprofloxacin and recovered well.

**Conclusions:**

This case report demonstrates the potential of *Chromobacterium* species as infectious agents in immunocompetent patients. It also indicates the existence of a novel species.

## Background

The genus *Chromobacterium* currently consists of fourteen species [1]. The most prominent species of the genus, *C. violaceum*, isolated and described in 1880 [2], was the only validly published species of the genus until the year 2007 [3]. *C. violaceum* has long been known to be prevalent in waters and soils of tropical and subtropical regions and has achieved great attention for the production of an insoluble dark, purple-colored pigment, violacein, which has interesting antimicrobial and other biotechnological properties [4–6]. Despite its metabolic flexibility and high prevalence in certain soils and waters, *C. violaceum* was seldom recognized to cause infections in humans [7]. Not until 1953 [8] was a death from infection by *Chromobacterium* reported; publications and case reports described skin, wound and systemic infections [8] in geographical areas most often located in tropical and subtropical regions, for example, South America and Southeast Asia. However, recently, infections in countries with moderate climate, such as Poland and Switzerland, have been reported [4]. The reported clinical manifestations of *C. violaceum* include fever, skin lesions, abdominal pain, abscesses, pneumoniae and sepsis, as well as endocarditis [9], which may progress to a fatal outcome [10]. The bacterium is often susceptible to ciprofloxacin but is known to be resistant to many clinically used antibiotic agents [11]. *C. violaceum* was believed to be the only *Chromobacterium* species pathogenic to humans [12] until 2013, when *C. haemolyticum* was reported to cause bacteremia in a previously healthy patient [13].

Here, we present a case of clinical infection with *Chromobacterium sp.*, presumably contracted close to a lake in northern Sweden. The patient was diagnosed with erysipelas and given recommended standard treatment with phenoxymethylpenicillin. Despite treatment, the patient clinically worsened, and growth in blood cultures was identified as *Chromobacterium sp*. (later identified as a presumably novel species). Treatment was changed to ciprofloxacin and clindamycin and, later, trimethoprim/sulfamethoxazole, after which, the patient recovered well.

## Case presentation

A 79-year-old man presented himself with a clinical picture that was assessed as a case of erysipelas. The patient’s medical history included atrial fibrillation, obstructive sleep apnea syndrome (OSAS), treatment for latent tuberculosis in 2021 and two episodes of transitional ischemic attack (TIA). No history of immunosuppression was recorded. The patient had travelled to Germany three weeks before his debut of symptoms but had never travelled outside of Europe during his lifetime.

**Fig. 1:**
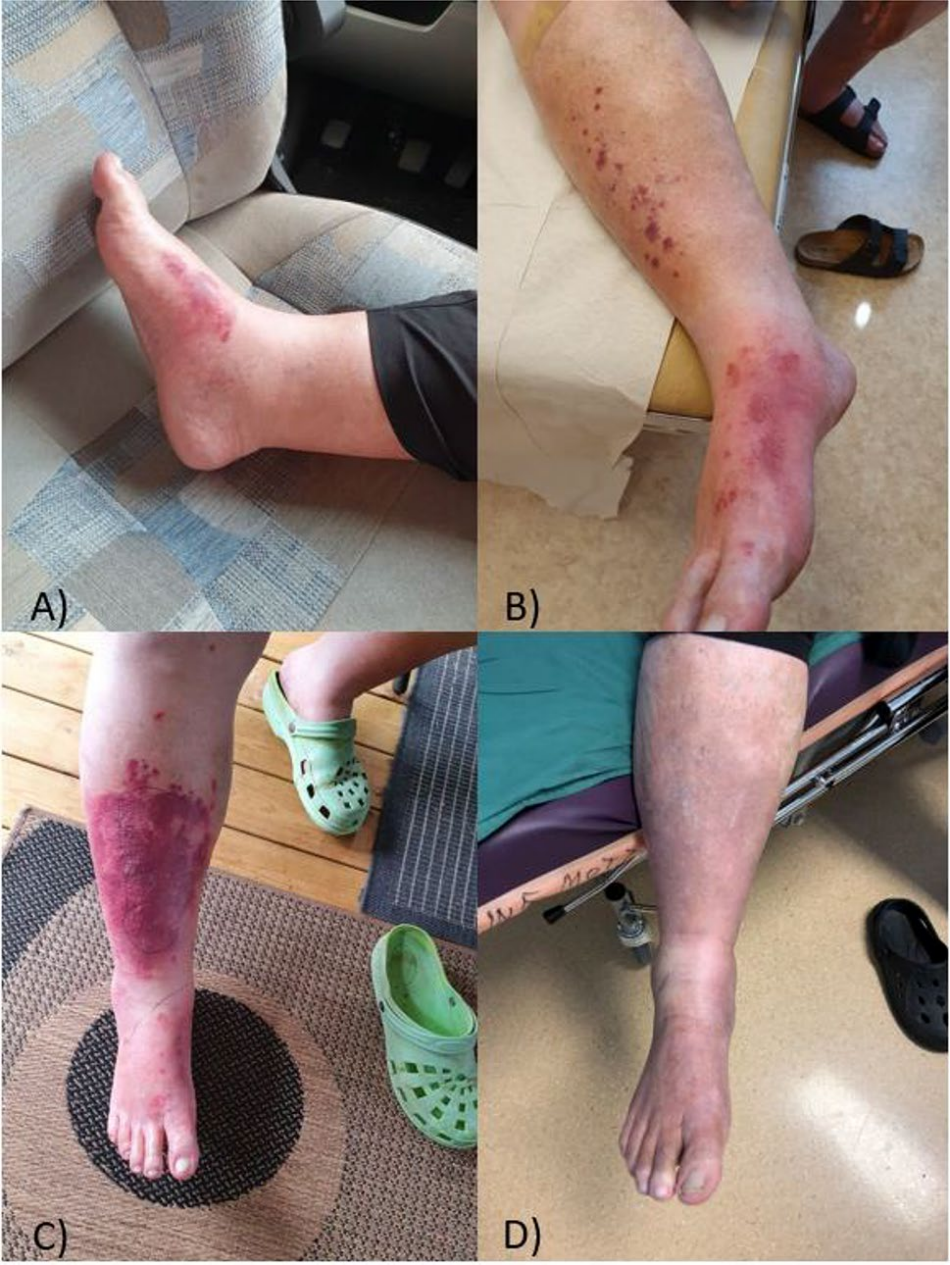
Timeline of the clinical presentation of the infection. **A**) Day 1 of symptoms, hours after the bite of a horsefly. **B**) Day 2 of symptoms, spreading of inflammation and rashes **C**) Day 3 of symptoms. After approximately 24 h of PcV treatment and 3 g of benzylpenicillin. **D**) 17 days after discharge with peroral antibiotics (see above)

Day 1 of symptoms (2022-06-30), the patient was visiting a lake in northern Sweden, close to the Swedish town, Jokkmokk, where he was bitten by a horsefly (*Tabanidae* family) on his right foot. Hours afterwards, the patient experienced redness, swelling and pain in the skin in association with the wound (Fig. 1A).

Day 2, the patient became febrile, and the area of inflammation had expanded proximally to the lower part of the same leg with erythematous rashes (Fig. 1B). The patient subsequently went to a primary health care center, where he was prescribed phenoxymethylpenicillin (PcV) for the case of erysipelas, an infection usually caused by *Streptococcus pyogenes/Streptococcus dysgalactiae* [14]. Later the same day, the patient went to the emergency clinic at Sundsvall Hospital with more severe symptoms, where a C-reactive protein (CRP) level of 91 mg/L was observed in blood samples. The clinical assessment at the hospital was, once again, erysipelas. Blood cultures were taken, and treatment was started with 1 dose of 3 g benzyl-penicillin given intravenously. The patient was sent home with continued phenoxymethylpenicillin treatment of 1 g x3.

Day 3, blood cultures returned positive, showing Gram-negative bacteria, and the patient was contacted for a new assessment. The clinical status of the leg had worsened, with an increased area of redness and swelling (Fig. 1C). CRP had increased to 238 mg/L and leukocyte count to 26 × 10^9^/L (reference: 3.5–8.8 × 10^9^/L). The patient was given 2 g of ceftriaxone intravenously and sent home with ciprofloxacin 500 mg x2, in addition to the current phenoxymethylpenicillin treatment. The patient was also scheduled for an assessment at the Department of Infectious Diseases two days later and received instructions to seek the emergency clinic upon any sign of worsening symptoms.

Day 4, in line with given instructions, the patient sought the emergency department for increasing pain and redness in the leg. The patient was then admitted to the Sundsvall hospital as an inpatient of the clinic for Infectious Diseases, with a treatment regimen consisting of cefotaxime 2 g x3 intravenously and clindamycin 300 mg x3 orally. At admission, the patient had a blood pressure of 105/59, CRP of 285 mg/L and a temperature of 37.7 degrees Celsius.

Day 5, blood cultures were identified as *C. violaceum*. Susceptibility tests were completed: cefotaxime was discontinued in favor of ciprofloxacin, due to the resistance pattern; ciprofloxacin was given at two doses of 400 mg intravenously and one dose of 500 mg orally.

Day 6, the clinical picture had improved, with decreased swelling, redness, and pain, along with loss of fever and CRP of 105 mg/L. The patient was then discharged from the hospital with a treatment regimen consisting of ciprofloxacin 500 mg x2 and clindamycin 300 mg x3, combined with continuous follow-up at the department for Infectious Diseases at Sundsvall Hospital.

One week after discharge, the clinical picture was still improving and clindamycin was ended and, after another 2 days, ciprofloxacin was replaced by trimethoprim/sulfamethoxazole 800 mg/160 mg x2, due to drug-induced skin rash.

After 8 more days, the clinical picture of the patient’s leg was close to habitus (Fig. 1D). The patient experienced no further symptoms and antibiotic treatment was ended with scheduled follow-up for blood-cultures.

## Laboratory analysis

Approximately 10 mL of blood was collected in each of two aerobic (BACTEC FX Plus Aerobic/F) and two anaerobic (BACTEC Lytic/10 Anaerobic/F) bottles and loaded into BD Bactec ^TM^ FX blood culture system. Both anaerobic bottles signaled positive after 13 h and both aerobic bottles signaled positive after 19 h. The Gram-stain showed Gram-negative rods in all four bottles and cultures showed bacterial growth on all plates (blood, hematin, chromogen and BHIA (anaerobic bottles)). The cultures were analyzed, using matrix-assisted laser desorption ionization time-of-flight mass spectrometry (MALDI-TOF MS) (MALDI Biotyper ^®^, Bruker Corp.), wherein the results showed no reliable identification. The cultures were then analyzed, using Vitek2 Compact (bioMérieux), Gram-negative card, with the result: *C. violaceum*, very good id (94% probability). Susceptibility testing was performed, using EUCAST guidelines. Gradient test showed MIC values of cefotaxime 1.0 mg/L, piperacillin/tazobactam 1.0 mg/L, trimethoprim/sulfamethoxazole 0.064 mg/L (ratio 1:19), ciprofloxacin 0.002 mg/L, gentamicin 4 mg/L and meropenem 0.064 mg/L. Due to the rarity of cases with *C. violaceum*, the isolate was sent to the Culture Collection University of Gothenburg (CCUG), in Gothenburg, Sweden for 16S rRNA gene sequence determination and identification analysis. The method used for DNA extraction, targeted PCR-amplification of the 16S rRNA genes and sequence determination and analysis is described in detail by Jaén-Luchoro et al. [15]; the sequence has been submitted to the National Center for Biotechnology Information (NCBI) and is available under the accession number OP750449. The 16S rRNA gene sequence of the strain (designated as CCUG 76385) collected from the patient, exhibited a 99.6% sequence similarity with the 16S rRNA gene sequence of the type strain of *C. amazonense* and a 99.8% sequence similarity with the type strain of the recently described *C. sinusclupearum* [16]. The sequencing was completed with a phenotypical description of smooth, violet-colored colonies.

## Discussion and conclusions

We have presented a clinical case of septicemia caused by *Chromobacterium sp.* in Sweden. The 16S rRNA gene sequence similarities of 99.8% and 99.6% to the type strains of *C. sinusclupearum* and *C. amazonense* (respectively) suggested the identity of *Chromobacterium sp*. CCUG 76385 to be likely one of the two species. The 16S rRNA gene sequence similarity of *Chromobacterium sp*. CCUG 76385 to *C. violaceum* was below the 98.7% similarity ‘cut-off’ expected for species-level identification [17]. However, a subsequent determination and analysis of the whole genome sequence indicates that CCUG 76385 is a strain of neither *C. sinusclupearum* nor *C. amazonense* but, in fact, most likely representing a novel species of *Chromobacterium* (data not shown). This determination can explain why MALDI-TOF MS analyses failed to provide a reliable identification (“no reliable id”). This determination would also explain why the Vitek 2 analysis only managed to provide an approximate identification of *C. violaceum* with 94% probability, given the limitations of commercial databases.

Interestingly, the CCUG documented another, unrelated case of *Chromobacterium sp*., isolated from a patient’s blood culture in 2016. The isolate (CCUG 69338) showed a 16S rRNA gene sequence identical (100% similarity) to that of CCUG 76385. These two cases suggest that at least one novel species of *Chromobacterium*, present in different geographical locations in Sweden, have caused infections similar to cases of infection by *C. violaceum* and *C. haemolyticum* [13].

An interesting aspect of this case is the bite of the horsefly just hours prior to presentation of symptoms. The patient described the increasing symptoms of swelling, redness and pain that furthermore culminated into fever, originating from the site of the bite. Transmission of diseases via horseflies do occur in medicine and veterinary health care [18]. Since horseflies occasionally may act as a vector for parasitic, viral, and bacterial infections, one should take into consideration the correlation of the time and location of the bite to the time and location of the symptoms. Furthermore, species of *Chromobacterium* have been identified in the midgut of mosquitos [19], indicating that transmission of *Chromobacterium* spp. by insects may be possible. Since species within *Chromobacterium* genus has been sampled from rivers (e.g., *C. amazonense*) [20], it might also be reasonable to assume that the current pathogen might be present in the lake and/or surrounding area. One might also speculate that the horsefly could have facilitated transmission via penetration of the skin barrier so that the inoculation was performed through contact with soil or water afterwards. Moreover, one may consider the role of the horsefly to be a direct transmitter of the pathogen from contact with the water to the skin of the patient.

As discussed previously, the genus *Chromobacterium* has long been known to be prevalent in tropical and subtropical regions [4]. However, its presence in northern Sweden, where the climate is markedly different, is intriguing. The metabolic flexibility of the genus, with isolations from new geographical locations, supports the probability of *Chromobacterium* spp. being more globally widespread than previously thought.

This case report displays several interesting factors. Firstly, it is significant that a clinical infection, obtained in the northern part of Sweden, is presented by a previously unidentified *Chromobacterium* species in a climate unusual for reported infections caused by species of the *Chromobacterium* genus. Secondly, the clinical value is not insignificant, since infection with *C. violaceum* has been spotted increasingly in temperate regions and the pattern for other *Chromobacterium* spp. may be similar [4]. Finally, the clinical picture may be misinterpreted as a case of erysipelas caused by *Streptococcus pyogenes/Streptococcus dysgalactiae*, which may result in an unfavorable outcome, due to inappropriate treatment regimen, as the initial management in this case demonstrated. This case report may guide future cases to more immediate correct treatment, since the subsequent treatment regimen was successful.

For future research, the genome sequence of strain CCUG 76385 is being analyzed in detail in preparation for describing a new species of *Chromobacterium*.

## Data Availability

The dataset generated from 16S rRNA gene sequence analysis is available at the National Center for Biotechnology Information (NCBI) under the accession number OP750449. Dataset regarding clinical information is not publicly available due to secrecy measures.

## Abbreviations

CCUG: Culture Collection University of Gothenburg
CRP: C-reactive protein
MALDI-TOF MS: matrix-assisted laser desorption ionization time-of-flight mass spectrometry
MIC: minimum inhibitory concentration
NCBI: National Center for Biotechnology Information
OSAS: obstructive sleep apnea syndrome
PCV: phenoxymethylpenicillin
TIA: transitional ischemic attack
